# Anaerobic Dechlorination by a Humin-Dependent Pentachlorophenol-Dechlorinating Consortium under Autotrophic Conditions Induced by Homoacetogenesis

**DOI:** 10.3390/ijerph16162873

**Published:** 2019-08-11

**Authors:** Mahasweta Laskar, Takanori Awata, Takuya Kasai, Arata Katayama

**Affiliations:** 1 Graduate School of Engineering, Nagoya University, Nagoya 464-8603, Japan; 2 National Institute for Land and Infrastructure Management, Tsukuba 305-0804, Japan; 3 Institute of Materials and Systems for Sustainability, Nagoya University, Nagoya 464-8603, Japan

**Keywords:** autotrophic, H_2_, CO_2_, humin, acetogens, reductive dechlorination, homoacetogenesis

## Abstract

Anoxic aquifers suffer from energy limitations due to the unavailability of organic substrates, as dictated by hydrogen (H_2_) for various electron-accepting processes. This deficiency often results in the accumulation of persistent organic pollutants, where bioremediation using organic compounds often leads to secondary contamination. This study involves the reductive dechlorination of pentachlorophenol (PCP) by dechlorinators that do not use H_2_ directly, but rather through a reduced state of humin—a solid-phase humic substance—as the extracellular electron donor, which requires an organic donor such as formate, lactate, etc. This shortcoming was addressed by the development of an anaerobic mixed culture that was capable of reductively dechlorinating PCP using humin under autotrophic conditions induced by homoacetogenesis. Here, H_2_ was used for carbon-dioxide fixation to acetate; the acetate produced was used for the reduction of humin; and consequently used for dechlorination through reduced humin. The 16SrRNA gene sequencing analysis showed *Dehalobacter* and *Dehalobacterium* as the possible dechlorinators, while *Clostridium* and *Oxobacter* were identified as the homoacetogens. Thus, this work contributes to the development of an anaerobic consortium that balanced H_2_ dependency, where efficiency of humin reduction extends the applicability of anaerobic microbial remediation in aquifers through autotrophy, syntrophy, and reductive dechlorination.

## 1. Introduction

Aquifer contamination by persistent organic pollutants (POPs) is a matter of serious concern considering biogeochemical and atmospheric processes as drivers for the magnification of this issue to a global scale, impacting water, soil, and also the food chain [1,2,3,4]. The anoxic niche in aquifers harbors different types of microorganisms in an intertwined network, mainly dependent on hydrogen (H_2_) as the donor for different electron-accepting processes (e.g., NO_3_^−^, Mn(IV), Fe(III), SO_4_^2−^, CO_2_) for their growth and metabolism. These H_2_-utilizing microbial reactions in turn make anaerobic bioremediation—especially anaerobic dehalogenation—a panacea for aquifer contamination removal [5,6]. *Dehalococcoides* is one such H_2_-utilizing dehalogenating microorganism (organohalide respirer) that has been found and characterized [7].

Pentachlorophenol (PCP), a POP currently listed under Stockholm Convention because of its carcinogenic and toxic effects, has received a great deal of attention as early as the 1970s, especially from its use as a pesticide in agricultural and wood preservation sectors [8,9,10]. In the anaerobic bioremediation or natural attenuation of PCP, reductive dechlorination is carried out by dechlorinators, where the chlorine atoms are substituted with hydrogen atoms (electrons) [11]. However, low growth or bioenergetics by such microorganisms is reflected by the high redox potential (+399 mV at pH 7 for PCP/2,3,4,5-tetrachlorophenol using H_2_); substrate, and nutrient limitation; and even geological composition [12,13]. For such bioenergetics metabolism, humic substances in soil help in crossing the energy barrier by transfer of electrons between the microorganisms and the electron-accepting compounds [14,15].

The catalytic centers present in soil for extracellular electron transfer (EET) have been explored in studies using humin, the insoluble humic substance in soil at any pH, which behaves as a redox mediator in reductive PCP dechlorination by the transfer of electrons from formate-oxidizing bacteria to the dechlorinators [16]. The humin-dependent dechlorinators did not use H_2_ as the electron donor, but required a reduced humin through input of an organic electron donor such as formate, or via electrochemically reduced humin [17,18,19]. The requirement of an organic electron donor has also been observed in other microbial reactions using humin, such as debromination of tetrabromobisphenol-A [20]; iron reduction, nitrate reduction to ammonia [18]; and denitrification [21].

It is already known that the introduction of organic electron donors has secondary impacts on bioremediation, such as excessive biomass growth, fouling, etc., and this concern has been addressed by the dissolution of H_2_ gas in aquifers for bioremediation [22,23]. We previously demonstrated that co-culturing the denitrifying bacterium *Pseudomonas stutzeri* JCM20778 with the acetogenic bacterium *Sporomusa ovata* DSMZ 2662 ensured denitrification in the absence of organic substrate by the use of the inorganic compounds H_2_ and carbon dioxide for substrate production [24]. Acetogenic microorganisms (e.g., *Sporomusa ovata*, *Clostridium ljungdahlii*, *Moorella thermoacetica,* etc.) utilize H_2_ for the production of organic acids such as acetate and oxobutyrate from CO_2_ in a process known as homoacetogenesis [25,26]. This suggests the manifestation of a hydrogenotrophic bioremediation system in coexistence with other microorganisms in an environment where carbon dioxide is indigenously present. The application of this phenomenon to organohalide-contaminated aquifers bioremediation is both lucrative and challenging, owing to the varying H_2_ requirement, as well as the redox potential (−290 mV from CO_2_/acetate) between dechlorinators and acetogens [27,28]. Concerning on humin-dependent PCP reductive dechlorination, the effect of autotrophy has not been observed until now. In this study, we developed a humin-dependent PCP-dechlorinating consortium with acetogenic activity, and demonstrated PCP reductive dechlorination through acetate oxidation that was produced due to homoacetogenesis using the inorganic compounds H_2_ and carbon dioxide.

## 2. Materials and Methods 

### 2.1. Humin

Humin was prepared by a modified extraction method of the previous paper [16]. Air-dried Kamajima soil (600 g) was sieved with 2 mm mesh sieves. The sieved soil was mixed with 150 mL distilled water in a 250 mL centrifugation bottle and shaken by a mechanical shaker (Taitec TS-20, Tokyo, Japan) for 30 min. The bottle was kept upright for 7 s, and decantation was carried out in 30 s to remove larger mineral particles in the soil. The remaining soil was mixed and decanted again with 150 mL of distilled water in the same manner. The two-times decanted soil fractions were combined and subjected to the humin extraction procedure, consisting of a series of washings with mechanical shaking for 24 h with chemicals, as follows: twice with 150 mL of 0.1 N NaOH, four times with 2% HF; twice with 75 mL of 0.1 N NaOH, twice with 2% HF; and a final wash of four times with 0.1 N NaOH. The obtained humin was then washed twice with distilled water for 12 h. The obtained humin was neutralized at pH 7.0 ± 0.2 using 0.1 N HCl. The neutralized humin was then washed twice with distilled water with 12 h mechanical shaking. In all the washing steps, the centrifugation was carried out at 8000× *g* (Kubota 7780, Tokyo, Japan) for 15 min at 23 °C to separate humin as precipitate in the washing solutions. The obtained humin was freeze dried, ground finely using ceramic mortar, and subjected to the experiment.

### 2.2. Humin-Dependent PCP-Dechlorinating Culture (HMBC Culture)

The HMBC culture was obtained using 10 mM acetate as the sole electron donor and carbon source, with 1 g of humin inoculation of a PCP-dechlorinating culture grown on Kamajima soil using lactate as the electron donor and carbon source, which had been maintained in the laboratory [29]. The total 120 mL volume capacity of a serum glass bottle was filled with 50 mL of an anaerobic medium and sparged with a mixed gas ratio of 4:1 N_2_ and CO_2_ until pH 7.2 ± 0.2 was achieved. The bottle was capped with a Teflon-coated butyl rubber stopper and sealed with aluminum crimp. The headspace was flushed with mixed gas of N_2_ and CO_2_ (4:1) through an air filter with pores of 0.22 µm diameter for 30 min to expel remaining traces of oxygen. The culture was incubated at 30 °C in the dark for two weeks and transferred serially by 10-fold dilutions for maintenance until use. The medium consisted of: NH_4_Cl 1.0 g/L; CaCl_2_·2H_2_O 0.05 g/L; MgCl_2_·6H_2_O 0.1 g/L; K_2_HPO_4_ 0.4 g/L; NaHCO_3_ 4.0 g/L; CH_3_COONa 0.82 g/L; 1 mL of selenite-tungstate solution; 1 mL of trace elements solution SL-10 [30]; and 1 mL of resazurin (0.1 g in 100 mL pure water). Figure S1 shows the community structure of the HMBC culture.

### 2.3. Homoacetogenic Culture (HC Culture)

A HC culture had been maintained in the laboratory using a mineral medium by serial transfer of 5% (*v*/*v*) of the culture every 14 days of incubation. The mineral medium composition used was modified DSMZ 311a medium as follows: K_2_HPO_4_ 0.35 g/L; KH_2_PO_4_ 0.23 g/L; NH_4_Cl 0.5 g/L; MgSO_4_·7H_2_O 0.5 g/L; CaCl_2_·2H_2_O 0.25 g/L; NaCl 2.25 g/L; FeSO_4_·7H_2_O 0.002g/L; Yeast extract 0.1 g/L; NaHCO_3_ 4 g/L; 1 mL of Selenite-Tungstate solution; and 1 mL of Trace Elements Solution SL-10. The mineral medium was sparged by the mixed gas of N_2_:CO_2_ until pH neutrality, and the headspace was flushed with H_2_ and CO_2_ (4:1) for 40 min post inoculation. Figure S2 shows the community structure of the HC culture.

### 2.4. Humin-Dependent PCP-Dechlorinating Culture with Acetogenic Activity (M-CO Culture)

The M-CO culture was obtained by mixing the HMBC and HC cultures in the ratio of 2:1. The M-CO culture was maintained in a mineral medium by serial transfer of 10% (*v*/*v*) of the culture. The mineral medium composition (Medium Z) consisted of: K_2_HPO_4_ 0.4 g/L; NH_4_Cl 0.5 g/L; MgSO_4_. 7H_2_O 0.3 g/L; CaCl_2_·2H_2_O 0.15 g/L; NaCl 1.0 g/L; FeSO_4_·7H_2_O 0.002 g/L; NaHCO_3_ 4.0 g/L; 1 mL of selenite-tungstate solution; 1 mL of trace elements solution SL-10; and 1 mL of resazurin (0.1 g in 100 mL pure water). The medium was sparged with a mixed gas ratio of 4:1 N_2_ and CO_2_ until pH 7.2 ± 0.2 was achieved. Vitamin solution [20], 20 µM sodium pentachlorophenol (>90% purity, Fujifilm Wako Pure Chemical Corporation, Osaka, Japan), and Ti-NTA solution (0.20 mM) were added prior to the inoculation. The headspace was flushed with H_2_ and CO_2_ (4:1) post inoculation. 

The sterilization of the anaerobic medium for culture bottles HMBC, HC, and M-CO was carried out by steam sterilization at 121 °C for 20 min prior to the inoculation. The inorganic chemicals used in the study used Special Grade (JIS) chemicals, and were purchased from FUJIFILM Wako Pure Chemical Corporation, Osaka, Japan.

### 2.5. Effect of Conditions on the PCP Dechlorination in the Mixed Consortium M-CO

Effects of inoculation and humin were examined using the M-CO culture. The effect of sulfide was examined by adding Na_2_S into the M-CO-B culture, where 0.3 g/L of MgSO_4_. 7H_2_O in medium Z was replaced with MgCl_2_ 6H_2_O of the corresponding concentration (medium ZN). Sulfate and salinity effects were examined in the HMBC culture by spiking with 0.5 g/L Na_2_SO_4_ and 2.25 g/L NaCl, respectively. Effects of humin oxidation and H_2_ were examined in the M-CO-B culture. The experimental conditions are summarized in Table 1.

### 2.6. Oxidized Humin Preparation

The oxidation of humin was carried out electrochemically using twisted platinum electrodes as working and counter electrodes (0.8 mm in diameter and 1 m in length), and a Ag/AgCl as reference electrode (+263 mV vs. SHE (Standard Hydrogen Electrode), Fusheng Analytical Instrument Co., Shanghai, China). The redox potential of 4 g humin in 150 mL anaerobic medium was maintained at 0 mV vs. Ag/AgCl electrode for 18 h. The oxidized humin was filtered using filter paper No. 2 (Advantec, Tokyo, Japan), and >0.9 g (approx.) oxidized humin was transferred to the respective triplicate cultures studied. The oxidation, filtration, and transfer of humin to the culture bottles was carried out in a vinyl anaerobic chamber (Coy-7450000, COY, Grass Lake, MI, USA). 

### 2.7. Chemical and Headspace Analyses

PCP and metabolites were extracted and analyzed as described previously [29] using a GCMS QP2010 (Shimadzu, Kyoto, Japan) equipped with a DB-5ms column (J7W Scientific, Folsom, CA, USA). Chlorophenols with >90% purity (FUJIFILM Wako Pure Chemical Corporation, Osaka, Japan) were used as standards. Organic acids were analyzed by HPLC (Shimadzu LC-10AT, Kyoto, Japan) with a Puresil C18 reversed-phase column (Waters, Milford, MA, US) and a UV detector at 210 nm. The mobile phase was 0.1% H_3_PO_4_ which was previously filtered using 0.45-µm PTFE membrane (Omnipore™, Merck, Darmstadt, Germany). The samples were provided by filtering the culture using a 0.2-µm membrane (Omnipore™, Merck, Darmstadt, Germany). Glacial acetic and formic acids with >99% purity (Cica KANTO CHEMICAL CO., INC., Tokyo, Japan) and 50% sodium lactate solution (FUJIFILM Wako Pure Chemical Corporation, Osaka, Japan) were used as standards. H_2_, CO_2_, and CH_4_ in the headspace were measured by a GC-14B gas chromatograph equipped with thermal conductivity and flame ionization detectors (Shimadzu, Kyoto, Japan). Sampling was carried out using a 100 µL-volume pressure-lock PTFE syringe (VICI, Baton Rouge, LA, USA) using nitrogen as the carrier gas.

### 2.8. Microbial Community Structure

Microbial biomass was collected from the fifth generation, and the microbial DNA was extracted using a FastDNA SPIN kit for soil (MP Biomedicals, Japan, Tokyo, Japan). PCR amplification of the bacterial 16S rRNA gene was performed with a primer set for amplification of the V3–V4 region as follows: Pro341F (5′-CCT ACG GGN BGC ASC AG-3′) and Pro805R (5′-GAC TAC NVG GGT ATC TAA TCC-3′) [31]. The reaction mixtures contained 12.5 µL of KAPA HiFi HotStart Ready mix (KAPA Biosystems, Wilmington, MA, USA), 2.5 µL of Pro341F and Pro805R primers (2 µM each), and 5 µL of template DNA (5 ng/µL). The PCR condition was as follows: initial activation at 94 °C for 30 s, followed by 10 cycles at 94 °C for 10 s, 60 °C for 30 s, 72 °C for 30 s, followed by 10 cycles at 94 °C for 10 s, 59 °C for 30 s, 72 °C for 30 s, followed by 10 cycles at 94 °C for 10 s, 58 °C for 30 s, 72 °C for 30 s, and final extension at 72 °C for 4 min. The PCR products were purified using the AMPure XP kit (Beckman Coulter Genomics Inc., Brea, CA, USA) according to the manufacturer’s instructions. The PCR products were confirmed using a 1% agarose gel. The concentration of purified DNA was determined using a QuantiFluor dsDNA System (Promega Corporation, Fitchburg, WI, USA). Purified DNA was sequenced using a Miseq platform with a Miseq reagent kit v3 (600 cycle, Illumina Inc., San Diego, CA, USA). A chimera check was performed for the base sequences of each read obtained from the analysis using USEARCH v6.1 [32]. Reads with more than 97% sequence similarity were classified into the same operational taxonomic unit (OTU), and OTU picking and cluster analysis were performed in QIIME 1.8 [33]. OTUs were identified using the Greengenes database (ver. 13_8) [34].

## 3. Results

### 3.1. Dechlorination of PCP by M-CO Culture 

**Figure 1:**
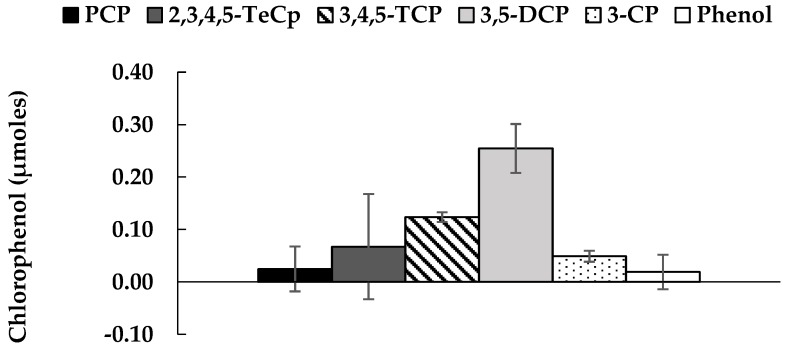
Remaining pentachlorophenol and the produced chlorophenols as the metabolites in the M-CO culture after two weeks of incubation. The values are the average of triplicate samples across three generations (nine bottles) with standard errors shown as vertical bars. 2,3,4,5-TeCP: 2,3,4,5-tetrachlorophenol; 3,4,5-TCP: 3,4,5-trichlorophenol; 3,5-DCP: 3,5-dichlorphenol; and 3-CP: 3-chlorophenol.

**Figure 2:**
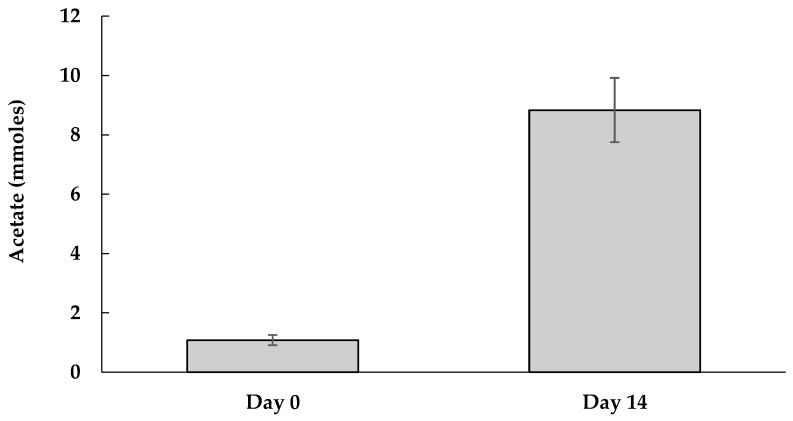
Increase of amount of acetate between Day 0 and Day 14 across three generations of the M-CO culture. The values are the average of the acetate amount measured for the respective triplicates of three generations (nine bottles), with standard errors shown as vertical bars.

**Figure 3:**
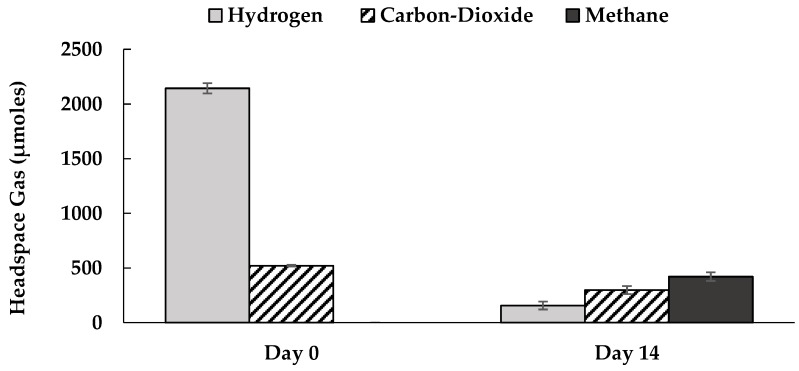
Changes in the composition of headspace gas between Day 0 and Day 14 for the M-CO culture. The bar graphs represent the average of triplicates across three generations (nine bottles), with standard errors shown as vertical bars.

Figure 1 shows PCP’s reductive dechlorination by the M-CO culture under autotrophic conditions after two weeks of incubation. The most prominent metabolites were 3,5-dichlorphenol (3,5-DCP) and 3,4,5-trichlorophenol (3,4,5-TCP), followed by 2,3,4,5-tetrachlorophenol (2,3,4,5-TeCP), 3-chlorophenol (3-CP), and occasionally phenol. The M-CO culture used H_2_ as the sole electron donor, and HCO_3_^−^/CO_2_ was the only carbon source; where bicarbonate (HCO_3_^−^) was added as a buffer for the medium, and the headspace was flushed with a mixed gas composed of H_2_ and CO_2_ (4:1). The mixed microbial consortium in the M-CO culture was developed by mixing the humin-dependent PCP-dechlorinating HMBC culture with the homoacetogenic CO_2_-fixing HC culture in the ratio of 2:1. Figure 2 shows the homoacetogenic activity observed in the M-CO culture. The acetate amount detected at Day 0 was a result of carry over by 10% (*v*/*v*) culture transfer, while acetate corresponding to Day 14 showed an increase from Day 0. The acetate production after two weeks of incubation agreed with the corresponding decrease of H_2_ and CO_2_ in the headspace, as shown in Figure 3. Methane generation was also observed in the headspace after two weeks of incubation. As PCP dechlorination was not observed in absence of the inoculum (basal condition) (Figure S3), PCP dechlorination under autotrophic condition was recognized due to microbial activity. Dechlorination activity was not observed in the cultures without humin (namely, the CO and HC-PCP cultures) (Figure S3), which indicated the dependency of this mixed consortium on humin for dechlorination activity. Note also that the dechlorination inactivity in the CO culture occurred despite external H_2_ addition, and acetate at a concentration larger than 1 mM present initially as carry over due to culture transfer. This suggested that the dechlorinating consortium here was unlike the typical organohalide respirers such as *Dehalobacter restrictus* or *Dehalococcoides*, which directly utilize H_2_ as electron donor and acetate as carbon source [35]. 

### 3.2. Inhibitory Factors in the M-CO Culture

**Figure 4:**
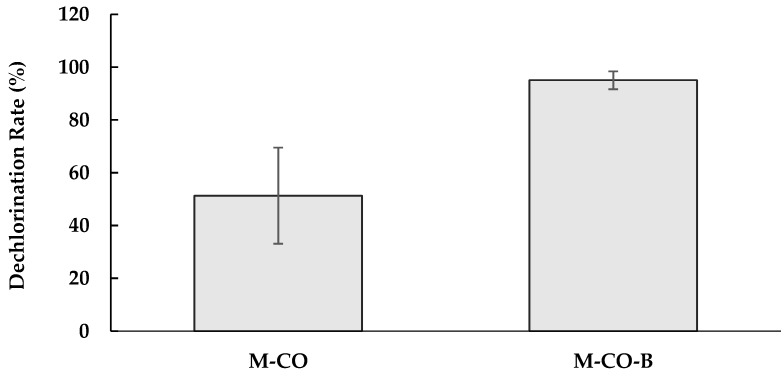
Dechlorination rate (%) in the M-CO and M-CO-B cultures after two weeks of incubation. The bar graphs represent the dechlorination rate calculated based on total organic chloride dechlorinated between Day 0 and Day 14 of incubation as a measure of dechlorination. The data represent the mean of triplicate samples, with standard errors shown as vertical bars. There was a significant difference between the dechlorination rates of the two cultures (*p* < 0.01).

The M-CO culture showed less dechlorinated metabolites in comparison to the metabolites observed for the parent dechlorinating HMBC culture (namely, 3-CP and phenol) (Figure S4), suggesting inhibitory effects. The blackish precipitation for the M-CO culture (Figure S5) indicated ferrous sulfide precipitation, where the sulfate concentration of 1.21 mM as sulfate in the medium Z exceeded the critical concentration of 100 µM producing the precipitation [14]. To examine the inhibitory effect due to sulfate reduction, the M-CO-B culture was provided in the medium ZN, where MgSO_4_·7H_2_O in medium Z was replaced with MgCl_2_·6H_2_O. Figure 4 shows the higher PCP dechlorination rate under autotrophic conditions in the M-CO-B culture than in the M-CO culture. The dechlorination metabolites for M-CO-B culture were 3-CP and phenol only. The effect of sulfide was further examined by adding 1.21 mM Na_2_S to the M-CO-B culture (i.e., MCO-Na2S culture). PCP dechlorination was not observed at all in the culture MCO-Na2S, indicating sulfide toxicity to be primarily the reason for the lower activity of the M-CO culture.

The inhibitory effects of sulfate and salinity were also tested for the parent dechlorinating HMBC culture. The dechlorinating culture contained 10 mM acetate as the electron donor, and H_2_ was not added externally. Addition of Na_2_SO_4_ to the HMBC culture (i.e., PCP-Sulfate culture) significantly inhibited PCP dechlorination, as shown by the lesser quantities of dechlorinated metabolites 2,3,4,5-TeCP, 3,4,5-TCP, 3,5-DCP, and 3-CP (Figure S6). Sodium chloride (NaCl) showed an even higher inhibitory effect for the HMBC culture (i.e., PCP-Sal culture) where 2,3,4,5-TeCP and 3,4,5-TCP (Figure S7) were the only observed metabolites. However, as NaCl was present for both medium Z and medium ZN, the inhibitory effect in the M-CO culture was not considered due to salinity, but sulfate reduction alone.

### 3.3. Role of Humin in Reductive Dechlorination

**Figure 5:**
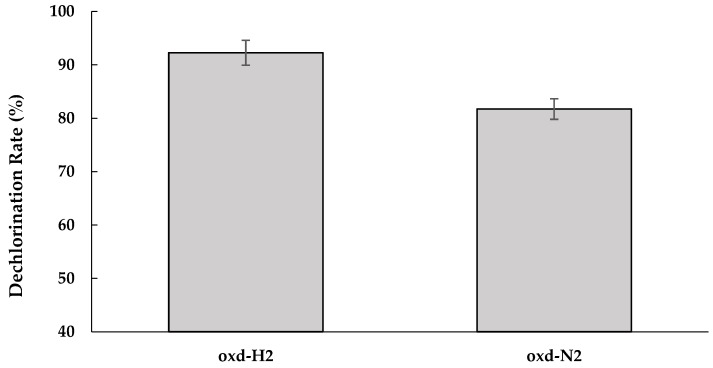
The effect of H_2_ on the dechlorination rate (%) after two weeks of incubation, shown in the cultures oxd-H2 and oxd-N2. The bar graphs represent the dechlorination rate calculated based on total organic chloride dechlorinated between Day 0 and Day 14 of incubation as a measure of dechlorination. The data represent the means of triplicate samples, with standard errors shown as vertical bars. There was a significant difference between oxd-H2 and oxd-N2 (*p* < 0.05).

The role of humin was examined in the M-CO-B culture using oxidized humin at 0 mV (vs. the Ag/AgCl electrode) under conditions with and without the external addition of H_2_ to the headspace (i.e., the oxd-H2 and oxd-N2 cultures, respectively). Figure 5 shows higher dechlorination activity for the culture oxd-H2 than the culture oxd-N2, implying that externally added H_2_ acted as an electron donor for dechlorination by this mixed consortium. This was also supported by acetate detection (57.95 µmoles) only for the culture oxd-H2, but not for the culture oxd-N2. Lower, but still observed, dechlorination activity for the culture oxd-N2 was considered to be influenced by the carry-over acetate due to the inoculum transfer, as the theoretical requirement for complete dechlorination of 20 µM PCP is only 25 µM of acetate, as calculated from Equations (1) and (2) [36,37]:

(1) $$2CO_{2}+4H_{2}↔CH_{3}COOH,$$

(2) $$C_{6}Cl_{5}OH+5H_{2}\to C_{6}H_{5}OH+5HCl.$$

### 3.4. Microbial Composition of M-CO Culture

**Figure 6:**
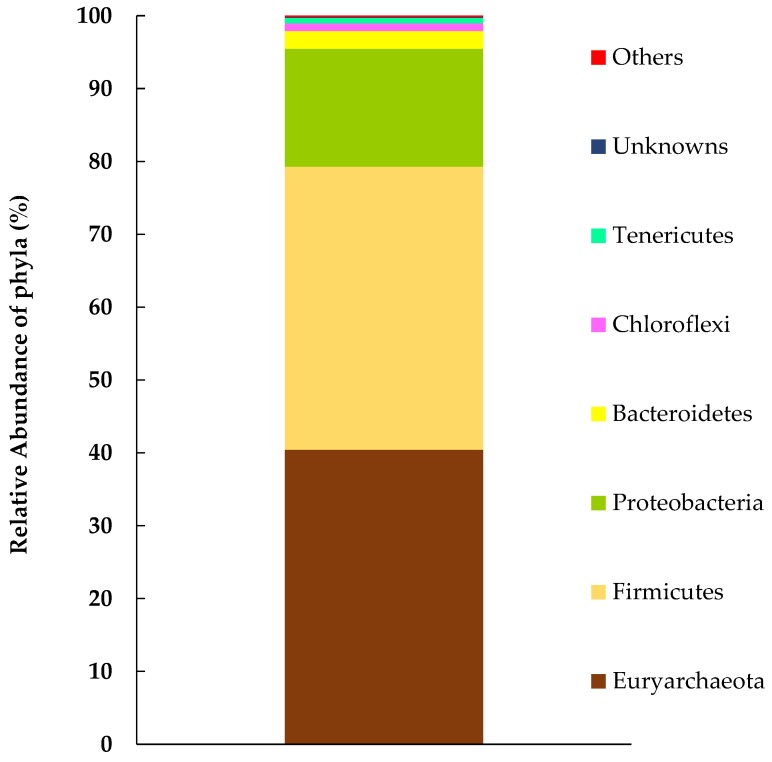
Microbial composition as identified by 16SrRNA gene sequencing for the M-CO culture. The major population identified for the phyla consisted of 40% Euryarchaeota, 39% Firmicutes, and 16% Proteobacteria. Others include phyla with relative abundance less than 0.5% (namely, spirochaetes, Crenarchaeota, Cyanobacteria, Actinobacteria, OD1, SAR406, and Thermotogae).

Figure 6 shows the microbial community structure of the M-CO culture. The major microorganisms belonged to the phyla Euryarchaeota, Firmicutes, Proteobacteria, Bacteroidetes, and Chloroflexi. Euryarchaeota were mostly represented by the genus *Methanobacterium*, which utilize H_2_ and CO_2_ for methane production. The genera *Methanoculleus* and *Methanomassiliicoccus* were also detected as Euryarchaeota. *Dehalobacter* and *Dehalobacterium* were detected as the probable dechlorinating bacteria [35,37]. The acetate-producing activity from CO_2_ is characterized by the bifunctional and oxygen sensitive enzyme complex of carbon-monoxide dehydrogenase (CODH) and acetyl-CoA synthase (ACS) [38], which can be distributed into diverse groups: methanogenic archaea, and acetogenic bacteria including Firmicutes, Deltaproteobacteria, and Chloroflexi. Of these diverse groups, the microorganisms detected in the M-CO culture were clostridia: *Clostridium* and *Oxobacter* as the autotrophic acetogenic bacteria [39,40], and *Caloramator* as the possible heterotrophic acetogenic bacteria [41]. An OTU classified into Desulfovibrionaceae was identified as a potential sulfate reducer, and heterotrophic acetogen as well in the absence of sulfate [42,43]. *Geobacter* is known to have electroactive characteristics, is a utilizer of acetate as an electron donor [15], and was occasionally detected in the M-CO culture.

The Shannon diversity index [44] showed a diversity of 2.39 with an equitability of 0.24, emphasizing the richness of the phyla mentioned earlier. The Chao 1 Index [44] measured a total 85 OTUs for the observed 69 OTUs, which reflected approximately 24.08% influence by the minor microbial groups as influential. 

## 4. Discussion

The microbial reductive dechlorination of PCP under autotrophic conditions was achieved by the cultures M-CO and M-CO-B, the mixed cultures obtained from the humin-dependent PCP-dechlorinating HMBC culture, and the homoacetogenic HC culture. The significant difference in the PCP dechlorination rate between the cultures with and without H_2_ (cultures oxd-H2 and oxd-N2, respectively) clearly demonstrated that H_2_ functioned as the electron donor for anaerobic dechlorination. No dechlorination was observed for the culture CO, confirming that dechlorinating organisms present in mixed culture did not utilize H_2_ as the electron donor, and that the presence of humin was essential as the external electron mediator for the dechlorinators. Additionally, acetate production in the cultures M-CO and M-CO-B was a result of homoacetogenesis. In a comparison between the cultures oxd-H2 and oxd-N2, acetate production was observed only for the culture oxd-H2, which accentuated the need of acetate as the secondary electron donor for humin reduction, and its consequent use for EET. These suggested that homoacetogenesis together with humin’s electron-mediating ability marked the pathway for syntrophic association to accommodate microbial reductive dechlorination under autotrophic conditions. A key syntrophic link between the acetogens, the acetate oxidizers involved in humin reduction, and the humin-oxidizing dechlorinators was established. 

Table 2 shows the distribution of electrons from H_2_ used for different sinks (i.e., acetate production, methane generation, sulfate reduction, and PCP reductive dechlorination). The electron balance for the M-CO culture suggested that a significant portion of electrons from H_2_ were diverted towards methane generation and sulfate reduction. While electrons invested for acetate production were minimal, it was almost negligible for dechlorination. In comparison to the M-CO culture, the electron balance for the M-CO-B culture showed that the total H_2_ used exceeded the requirement of electrons spent for the respective sinks, demonstrating the presence of an unknown sink. The only plausible explanation for the unknown sink was humin, considering the characteristics of humin as a redox mediator under anaerobic conditions. This interpretation agrees with the electron balance showing a negative value in the culture oxd-N2, where PCP dechlorination was observed although no H_2_ was supplied as an electron donor. Here the negative value of electron balance showed the presence of an unknown electron donor for dechlorination and methane generation, most probably with humin acting as an electron donor. The addition of H_2_ (i.e., the culture oxd-H2) increased the PCP dechlorination rate, with a decreased dependency on the unknown electron donor, in addition to the production of acetate and methane. It should be also noted that although humin in the cultures of oxd-H2 and oxd-N2 was electrochemically oxidized at 0 mV vs. Ag/AgCl (i.e., +263 mV vs SHE) for 18 h, the oxidized humin was still active as an electron donor for PCP dechlorination and methane production. The redox center of humin as an electron mediator is considered difficult to be electrochemically oxidized. 

The direct sinks of H_2_ (electron) were for acetogenesis, methanogenesis, and sulfate reduction, while PCP dechlorination was the indirect sink via the external electron transfer of humin. The threshold concentration of H_2_ is known to be higher in the order of acetogenesis (>0.7 meeq as electron), methanogenesis (0.01–0.2 meeq), and sulfate reducers (0.002–0.02 meeq) [27,49]. Therefore, the decrease of H_2_ distribution rate for acetogenesis would be unavoidable because of the predominance of methanogenesis and sulfate reduction, especially after the H_2_ concentration decreased. Sulfate reduction (sulfide production) resulted in an inhibitory effect on the PCP dechlorination, as well as reduced the electron flow for PCP dechlorination. The methanogens and sulfate-reducing microorganisms can use both acetate and H_2_ as electron donors in general [50]. However, in the M-CO culture, *Methanobacterium* was detected as the major methanogen and *Desulfovibrionaceae* as the major sulfate-reducing microorganism, and they are both hydrogenotrophic [43,51]. Therefore, the acetate produced would be mainly utilized for the humin reduction, although other acetate-utilizing reactions cannot be discarded. The microbial community analysis suggested *Geobacter* as the possible acetate-utilizing humin reducer. Humin oxidizers other than dechlorinators have not been identified. Further study should be carried out to determine the microorganisms involved in the reduction and oxidation of humin, which has potential as a versatile electron mediator for various microbial reactions [18].

## 5. Conclusions

**Figure 7:**
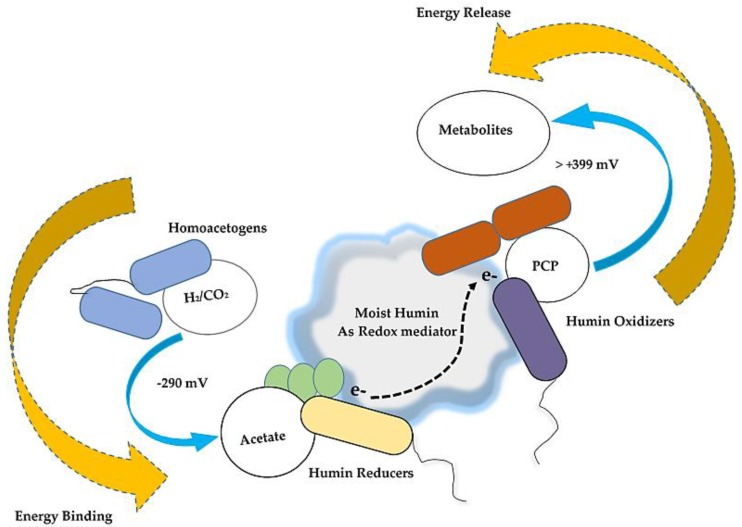
Electron transfer for reductive PCP dechlorination in an autotrophic environment. The figure describes the bioenergetic energy difference associated with autotrophic acetogens and dechlorinators. Here humin in its conductive state acts as the interface for electron transfer from acetate oxidizers (humin reducers) to humin oxidizers as the dechlorinators.

An anaerobic mixed culture achieved 20 µM PCP reductive dechlorination in the absence of any form of external addition of organic substrates as carbon source or energy. An autotrophic environment dictated by homoacetogenesis resulted in the establishment of reductive PCP dechlorination by the cultures M-CO and M-CO-B. Electrons provided as H_2_ were utilized as direct sinks for acetate production, methane generation, and sulfate reduction. The acetate produced was used for humin reduction by the humin reducers (the indirect sink), and facilitated external electron transfer to the dechlorinators for PCP dechlorination. Humin thus worked as an interface between two specific groups—the humin-reducers and the humin-oxidizers—and thereby maintained a steady balance of electron diversion from acetate to be used for PCP dechlorination, as shown in Figure 7. The results extended the applicability of such a humin-dependent dechlorinating culture for bioremediation when supplied with only H_2_ as an electron donor, constraining secondary pollution in anoxic aquifers. However, sulfate limitations have to be properly understood in such cases, and the re-routing of electrons towards methanogenesis needs to be addressed, which results in overall decreased efficiency for such systems. 

## Figures and Tables

**Table 1:** Experimental setup in this study. HC: homoacetogenic culture; HMBC: humin-dependent PCP-dechlorinating culture; PCP: pentachlorophenol.

Condition Name	Humin (1 g)	Humin State	Medium	Headspace Composition	Composition of Inoculum		Additional Treatment
					10% (*v*/*v*) HMBC	5% (*v*/*v*) HC	
M-CO	+	Intact	medium Z	H_2_/CO_2_	+	+	−
CO	−	−	medium Z	H_2_/CO_2_	+	+	−
HC-PCP	−	−	medium Z	H_2_/CO_2_	+	−	−
Basal	+	Intact	medium Z	H_2_/CO_2_	−	−	−
M-CO-B	+	Intact	medium ZN	H_2_/CO_2_	+	+	−
MCO-Na2S	+	Intact	medium ZN	H_2_/CO_2_	+	+	1.21 mM Na_2_S
oxd-H2	+	Oxidized	medium ZN	H_2_/CO_2_	+	+	−
oxd-N2	+	Oxidized	medium ZN	N_2_/CO_2_	+	+	−
PCP-Sulfate	+	Intact	medium PCP	N_2_/CO_2_	+	−	0.5 g/L NA_2_SO_4_
PCP-Sal	+	Intact	medium PCP	N_2_/CO_2_	+	−	2.25 g/L NaCl

**Table 2:** Distribution of H_2_ as milli electron equivalent (meeq) used for electron sinks in acetate production, methane production, sulfate reduction, and PCP dechlorination. *

Culture Name	Hydrogen Used	Electron Sink				Electron Balance **
		Acetate Produced	Methane Produced	Sulfate Reduction	Dechlorination	
M-CO	3.973	0.062	3.366	0.484	0.003	0.061
M-CO-B	3.082	0.067	2.576	-	0.010	0.429
oxd-H2	4.942	0.455	5.096	-	0.010	−0.619
oxd-N2	-	-	0.784	-	0.008	−0.776

* The calculation was based on the decrease in headspace H_2_ as an electron source, and the increase in the product amounts as electron sinks between Day 0 and Day 14. The solubility of the gas was calculated as per the Ostwald dilution law [45]. The calculation of electrons was based on the energetic reactions using hydrogen, as provided in Table S1 [12,25,46,47,48]. ** Positive values suggest the unknown sink, and negative values an unknown electron donor other than H_2_.

## Supplementary Materials

The following are available online at https://www.mdpi.com/1660-4601/16/16/2873/s1. Figure S1: Microbial composition of the dechlorinating HMBC culture based on 16SrRNA gene sequencing. Figure S2: Microbial composition of the homoacetogenic HC culture based on 16SrRNA gene sequence. Figure S3: Remaining PCP after two weeks of incubation with no chlorophenol metabolites detected for the cultures under different conditions except for headspace containing H_2_:CO_2_ (1:4). Figure S4: PCP-dechlorinated phenolic metabolites detected in the HMBC culture after two weeks of incubation, where 10 mM acetate was used as organic electron donor and carbon source. Figure S5: Blackish precipitation observed in the M-CO culture (medium Z with headspace of H_2_ and CO_2_ (4:1)) after two weeks of incubation. Figure S6: Remaining PCP and chlorophenol metabolites detected in the HMBC culture after two weeks of incubation when spiked with 1.217 mM sulfate (PCP-Sulfate culture in Table 1). Figure S7: Remaining PCP and chlorophenol metabolites detected in the HMBC culture spiked with 2.25 g/L NaCl (PCP-Sal culture in Table 1) after two weeks of incubation. Table S1: Hydrogenolytic reactions using different electron acceptors that may occur for M-CO and M-CO-B cultures.
