# Supplementary material for: Anaerobic Dechlorination by a Humin-Dependent Pentachlorophenol-Dechlorinating Consortium under Autotrophic Conditions Induced by Homoacetogenesis
Source: Int J Environ Res Public Health. 2019 Aug 11;16(16):2873. doi: 10.3390/ijerph16162873 (PMC6720667; doi:10.3390/ijerph16162873)
Supplement: Supplementary file 1 [file ijerph-16-02873-s001.pdf]

Supplementary Information for

Anaerobic dechlorination by a humin-dependent pentachlorophenol-dechlorinating consortium under autotrophic conditions induced by homoacetogenesis

Mahasweta Laskar[1], Takanori Awata[2], Takuya Kasai[1,3], Arata Katayama[1,3*]

[1] Graduate School of engineering, Nagoya University, Nagoya 464-8603, Japan

[2] National Institute for Land and Infrastructure Management, Tsukuba 305-0804, Japan

[3] Institute of Materials and Systems for Sustainability, Nagoya University, Nagoya 464-8603, Japan

* Correspondence: katayama.arata@nagoya-u.jp ; Tel.: +81-(0)52-789-5856

Number of pages: 6

Number of tables: 1

Number of figures: 7

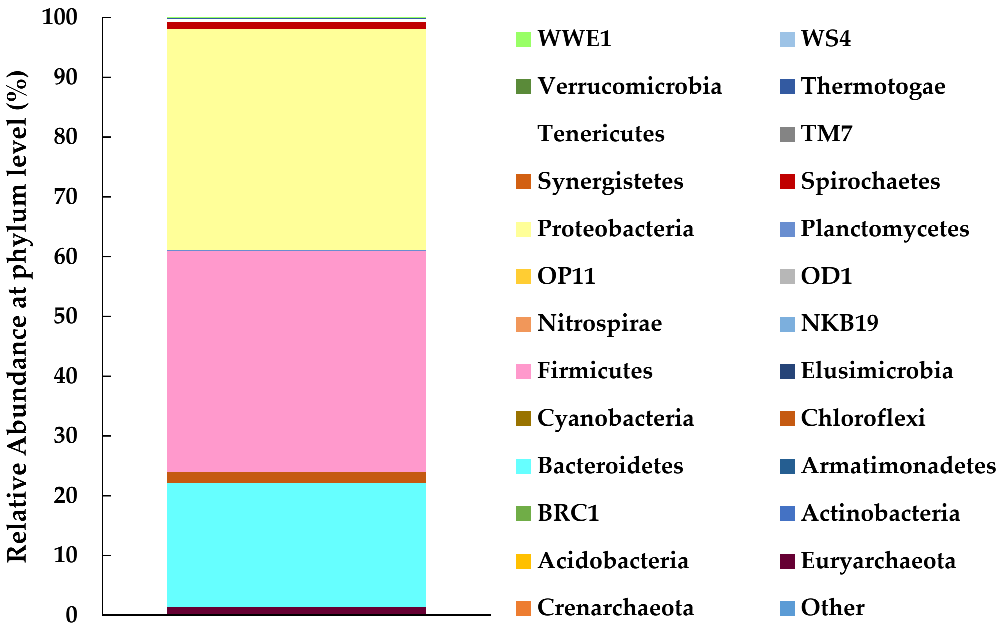

Figure S1. Microbial composition of the dechlorinating HMBC culture based on 16SrRNA gene sequencing. The major phyla belonged to *Proteobacteria* (37%), *Firmicutes* (>36%), *Bacteroidetes* (>20%) followed by less than 2% of *Chloroflexi*, *Spirochaetes*, and *Euryarchaeota*. The culture used 10mM acetate as an electron donor, or carbon source, and was incubated under anaerobic condition at 30°C for two weeks.

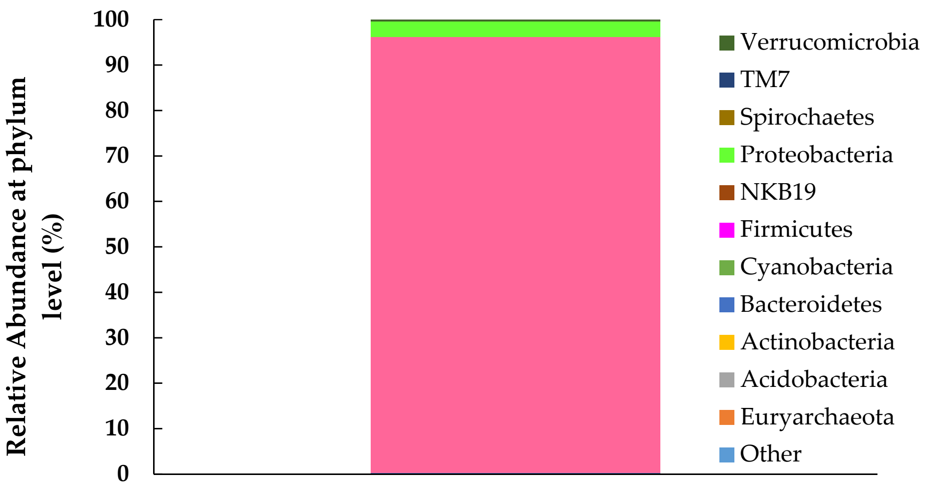

Figure S2. Microbial composition of the homoacetogenic HC culture based on 16SrRNA gene sequence. The major phyla belonged to *Firmicutes* (96 % approx) followed by *Proteobacteria* (<3.5 %). The headspace of the HC culture was flushed with H_2:CO_2 (4:1) post inoculation (5% v/v transfer), and incubated for two weeks at 30°C. The average acetate concentration over three generations after two weeks of the incubation was approximately 3 mM.

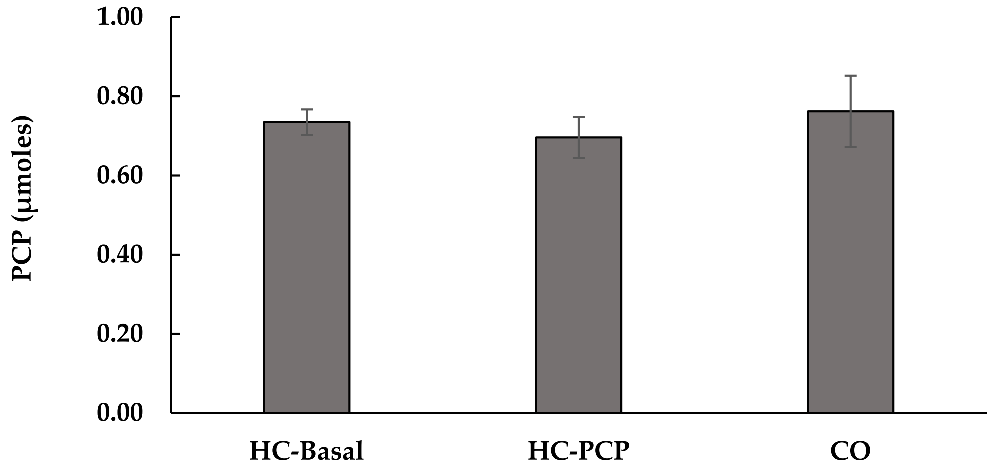

Figure S3. Remaining PCP after two weeks of incubation with no chlorophenol metabolites detected for the cultures under different conditions except for headspace containing H_2:CO_2: (1:4). The conditions of HC-Basal, HC-PCP, and CO cultures are summarized in Table 1. The data represents the average values of triplicates with standard deviations shown as vertical bars.

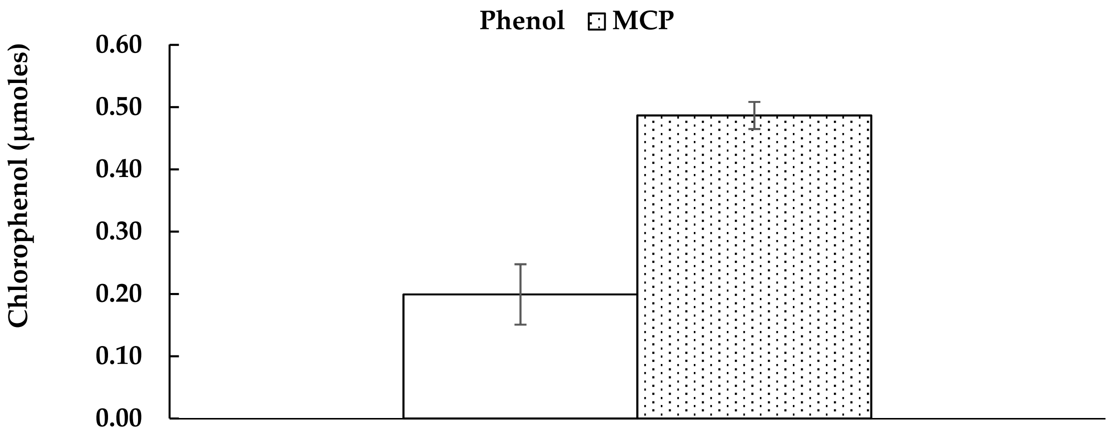

Figure S4. PCP dechlorinated phenolic metabolites detected in the HMBC culture after two weeks of incubation, where 10 mM acetate was used as organic electron donor, and carbon source. The values represent the duplicates mean with showing the difference by vertical bars.

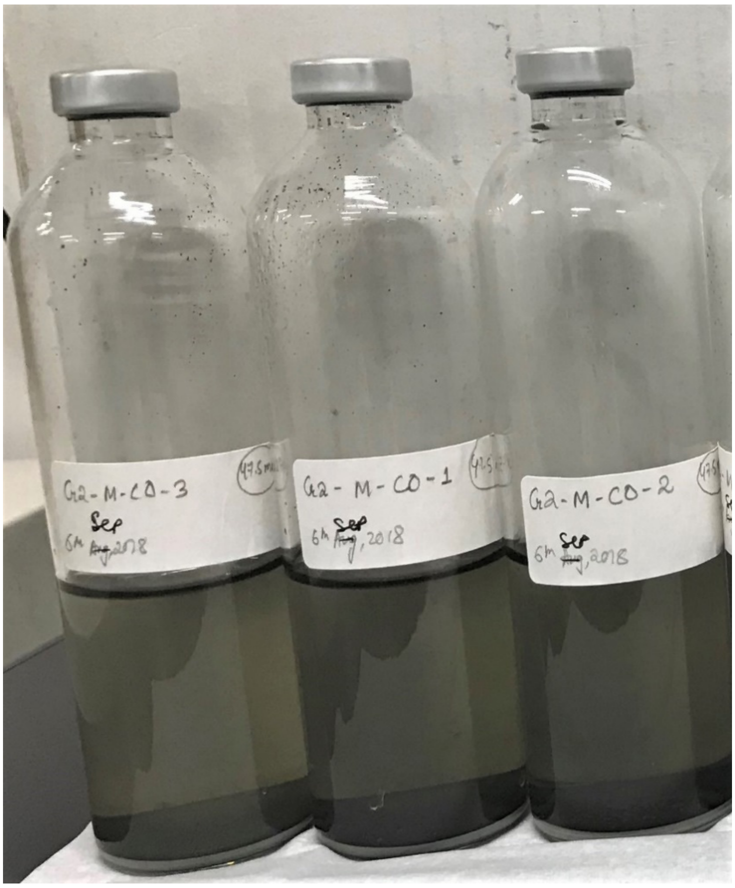

Figure S5. Blackish precipitation observed in the M-CO culture (medium Z with headspace of H_2 and CO_2 (4:1)) after two weeks of the incubation.

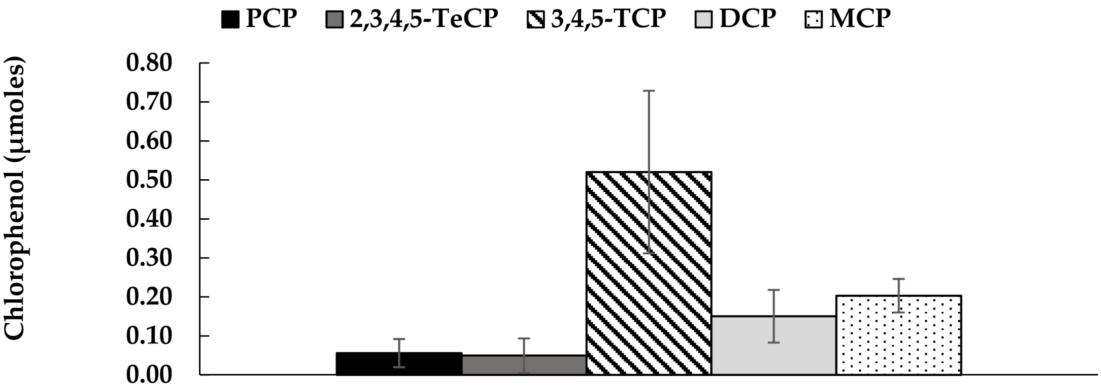

Figure S6. Remaining PCP and chlorophenol metabolites detected in the HMBC culture after two weeks of the incubation when spiked with 1.217 mM sulfate (PCP-Sulfate culture in Table 1). The data represents the average of triplicate samples with standard error shown as vertical bars.

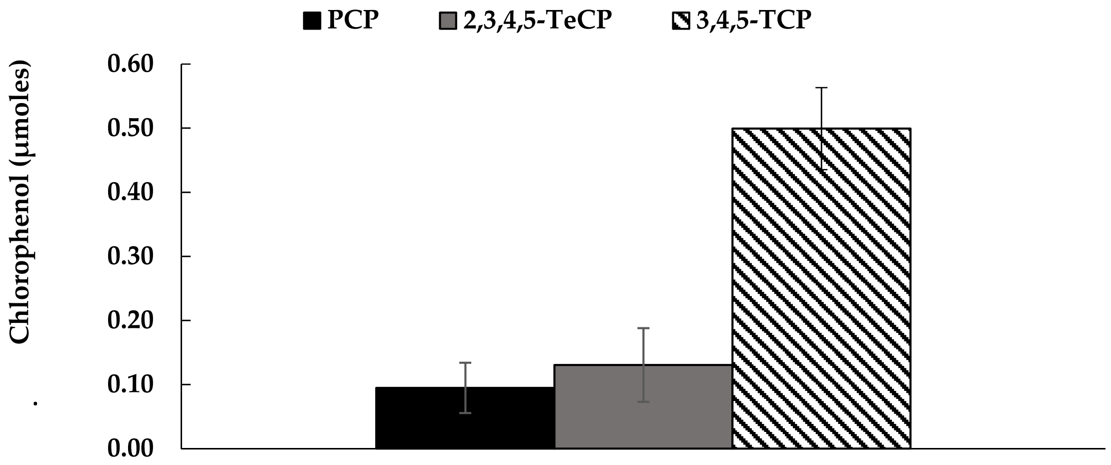

Figure S7. Remaining PCP and chlorophenol metabolites detected in the HMBC culture spiked with 2.25 g/L NaCl (PCP-Sal culture in Table 1) after two weeks of the incubation. The data show the mean values of duplicate samples with showing the difference by vertical bars.

Table S1. Hydrogenolytic reactions using different electron acceptors that may occur for the M-CO and M-CO-B cultures.

Redox Compound	Chemical Reaction	Gibbs free energy $\Delta G_o'$ (kj/mol)	Equation	References
CO_2/CH_3COOH	$2CO_2 + 4H_2 \leftrightarrow CH_3COOH$	-95	A	[1]
CO_2/CH_4	$CO_2 + 4H_2 \rightarrow CH_4 + 2H_2O$	-131	B	[2]
HCO_3^-/CH_4	$HCO_3^- + 4H_2 + H^+ \rightarrow CH_4 + 3H_2O$	-135.6	C	[3]
HCO_3^-/CH_3COO^-	$2HCO_3^- + 4H_2 + H^+ \rightarrow CH_3COO^- + 4H_2O$	-104.6	D	[3]
SO_4^{2-}/HS^-	$SO_4 + CH_3COO^- + 2H^+ \leftrightarrow HS^- + 2CO_2 + 2H_2O$	-57	E	[1]
SO_4^{2-}/HS^-	$SO_4 + 4H_2 + H^+ \leftrightarrow HS^- + 4H_2O$	-262.06	F	[4]
PCP/2,3,4,5-TeCP	$C_6Cl_5OH + H_2 \rightarrow C_6Cl_4OH_2 + HCl$	-167.9	G	[5]
2,3,4,5-TeCP/3,4,5-TCP	$C_6Cl_4OH_2 + H_2 \rightarrow C_6Cl_3OH_3 + HCl$	-161.7	H	[5]
3,4,5-TCP/3,5-DCP	$C_6Cl_3OH_3 + H_2 \rightarrow C_6Cl_2OH_4 + HCl$	-159.9	I	[5]
3,5-DCP/3-CP	$C_6Cl_2OH_4 + H_2 \rightarrow C_6ClOH_5 + HCl$	-149.7	J	[5]
3-CP/Phenol	$C_6ClOH_5 + H_2 \rightarrow C_6H_5OH + HCl$	-144.2	K	[5]

References:

1. Ragsdale, S.W.; Pierce, E. Acetogenesis and the Wood-Ljungdahl pathway of CO2fixation. *Biochimica et Biophysica Acta - Proteins and Proteomics* **2008**, *1784*, 1873–1898.

2. Ferry, J.G. CO Dehydrogenase of Methanogens. *Acetogenesis* **2012**, 539–556.

3. Oren, A. There Must be an Acetogen Somewhere. *Frontiers in Microbiology* **2012**, *3*, 2–3.

4. Ozuolmez, D.; Na, H.; Lever, M.A.; Kjeldsen, K.U.; Jørgensen, B.B.; Plugge, C.M. Methanogenic archaea and sulfate reducing bacteria co-cultured on acetate: Teamwork or coexistence? *Frontiers in Microbiology* **2015**, *6*, 1–12.

5. Dolfing, J.; Novak, I. The Gibbs free energy of formation of halogenated benzenes, benzoates and phenols and their potential role as electron acceptors in anaerobic environments. *Biodegradation* **2014**, *26*, 15–27.
